# The Influence of Music Reading on Spatial Working Memory and Self-Assessment Accuracy

**DOI:** 10.3390/brainsci14111152

**Published:** 2024-11-17

**Authors:** Michel A. Cara

**Affiliations:** Department of Pedagogy, Music Institute, Faculty of Philosophy and Education, Pontificia Universidad Católica de Valparaíso, Valparaíso 2340025, Chile; michel.cara@pucv.cl

**Keywords:** music reading, metacompetences, spatial working memory, computer-based neuropsychological testing, visuospatial capabilities, metacognititon, Corsi block-tapping test

## Abstract

Background/Objectives: Previous research has suggested that Western musicians, who generally demonstrate proficiency in reading musical scores, exhibit superior performance in visuospatial working memory tasks compared to non-musicians. Evidence indicates brain activation in regions such as the left inferior parietal lobe and the right posterior fusiform gyrus during music reading, which are associated with visuospatial processing. This study aimed to explore how musical training influences spatial working memory and to examine the relationship between self-assessment accuracy and cognitive performance. Methods: A visuospatial working memory test, the Corsi block-tapping test (CBT), was administered to 70 participants, including 35 musicians with experience in music reading and 35 non-musicians. CBT performances were compared between groups, controlling for sex and age differences using analysis of covariance. Participants were also asked to self-assess their visuospatial capabilities. Results: Musicians performed significantly better than non-musicians in the CBT and demonstrated greater metacognitive accuracy in evaluating their visuospatial memory capacities. A total of 46.34% of musicians who claimed good performance on the CBT did in fact perform well, in comparison with 14.63% of non-musicians. Sex influenced the outcomes of spatial working memory, while age did not significantly affect performance. Conclusions: This self-awareness of visuospatial capabilities reflects a form of metacompetence, encompassing reflective thinking and the ability to assess one’s cognitive skills. Furthermore, while differences in spatial working memory between musicians and non-musicians appear to be related to executive functions associated with general music practice, further investigation is needed to explore other potential influences beyond musical experience.

## 1. Introduction

Differences between musicians and non-musicians are often attributed to long-term music training, which induces both structural and functional neuroplastic changes in the brain. Rodrigues et al. [1] proposed three primary perspectives to understand the effects of music training on the human brain: (1) the art-producing effects of music on the brain, (2) specific alterations in the organization of the cerebral cortex, and (3) cognitive characteristics shaped by music training. Building on these perspectives, prior research has demonstrated that such neuroplastic adaptations are closely linked to enhanced cognitive functions, particularly in the realm of working memory, where musicians consistently exhibit superior performance. However, whether this training fosters a greater awareness of one’s cognitive abilities—potentially leading to what is termed “metacompetence”—remains an underexplored area of research.

This study seeks to address this gap by investigating not only how musical training influences spatial working memory but also how accurately musicians and non-musicians assess their cognitive performance. Specifically, we aim to explore whether systematic practice in music reading, framed within the context of expertise development, contributes to heightened self-awareness regarding the enhancement in general cognitive abilities. To gain deeper insights into the relationship between visuospatial cognition and music reading, we compared musicians with experience in reading music to non-musicians using a spatial working memory task. Our research seeks to answer the following key question: Does regular engagement in music reading contribute to a musician’s accuracy in evaluating their visuospatial capabilities?

### 1.1. Music Training and Visuospatial Working Memory

Working memory can be defined as a complex multi-component system that stores and processes information for temporary purposes [2]. This allows the brain to process new information linked to attention control, supported by a central executive component [3]. The phonological loop and the visuospatial sketchpad components store and handle verbal (speech and sound) and visuospatial information, respectively. The episodic buffer component functions as an interface between working and long-term memory and combines auditory and visual information. Given its integrative nature, working memory plays a crucial role in activities such as music training, which involves complex cognitive processes including auditory, visual, and motor skills [4]. Numerous studies have explored the potential causal relationship between music training and enhanced working memory capacity (see [5] for a review). However, this claim remains debated due to the predominance of cross-sectional study designs and the relative scarcity of longitudinal research [6]. Additionally, Vuvan et al. [7] stressed the need to determine whether this relationship is domain-general or domain-specific, specifically whether the observed benefits are confined to auditory memory (near transfer) or extend to broader working memory functions (far transfer).

Previous studies have further tested the influence of musical practice on visuospatial abilities. Talamini et al.’s [8] meta-analysis found that musicians consistently outperformed individuals with less musical training in tasks involving long-term memory, short-term memory, and working memory. However, the authors noted that while task type may mediate this relationship, the connection to visuospatial memory is less evident. Silas et al. [6] suggested that there is no direct relationship between visuospatial working memory and music training; instead, a domain-specific musical working memory mediates this relationship. However, the analysis did not identify a singular causal model, as initially anticipated. Other studies present contrasting findings, showing a visual advantage in adult musicians between 60 and 83 years old [9] or demonstrating that music training led to significant improvements in both visual and auditory memory in participants [10].

Bigand and Tillmann [11], after a revision of the work by Sala and Gobet [12], concluded that the far transfer from musical practice to other cognitive domains is plausible. While the effect sizes may appear relatively small, they argued that the possibility of a causal relationship cannot be entirely ruled out. Meta-analyses conducted by Cooper [13], Román-Caballero et al. [14], and Perez-Eizaguirre et al. [15] demonstrated that children, adolescents, and adults who undergo musical training show cognitive advantages in areas such as executive function, memory, and intelligence. These results reinforce the conclusions drawn by Bigand and Tillmann [11], but they also underscore the ongoing debate in the field, which often hinges on methodological concerns and a cautious approach in meta-analytical interpretations. Similarly, Sala and Gobet [16] noted that experimental studies incorporating longitudinal musical training have yielded mixed outcomes. Schellenberg [17] raised concerns about a tendency in the field to show unrestrained enthusiasm for neuroplasticity and to place excessive confidence in neuroimaging data, often overlooking insights from behavioral genetics and issues distinguishing causation from correlation in research on the effects of music training. Recent neuroimaging meta-analyses, including Pando-Naude et al. [18] demonstrated that music perception and production rely on similar brain networks, while music imagery uniquely activates parietal and motor regions. Criscuolo et al. [19] further highlighted that musical expertise corresponds to specific bilateral cortico-subcortical neuroanatomical and functional distinctions, suggesting that musical training offers a rich, multisensory experience that engages various neurocognitive functions. However, they cautioned that methodological inconsistencies—such as sample variability and overlooked background factors—remain significant challenges in research on the effects of music training on the brain.

Music reading, a dual task involving both reading and execution, relies heavily on working memory to enrich and transform information, ensuring coherence in the musical discourse [20]. This process involves linking and integrating old information with new [21]. Reading music systematically could then strengthen the development of visuospatial abilities and visuospatial resolution [22] as a result of long-term music practice. Enhanced visuospatial capabilities in musicians seem to be related particularly to efficient attentional processes [23,24,25], prolonged experience or activities related to playing a musical instrument [26], or even chunking abilities “to facilitate perceptual encoding” during a music score change detection task in comparison with non-musicians [27]. Sluming et al. [28] reported behavioral and neurofunctional evidence of differences between orchestral musicians and non-musicians in the performance of a visuospatial trial involving two- and three-dimensional tasks. The authors suggested that non-musical visuospatial cognition can be enhanced as a result of practicing sight-reading, defined as the capacity to perform written music without prior rehearsal. Rodrigues et al. [29] found no significant differences between musicians and non-musicians in a visual task involving the memorization of eight figures. However, musicians exhibited faster reaction times, which the authors interpreted as indicative of enhanced visual attention abilities. Similar results were reported by Hansen et al. [30], who found no differences between amateur musicians, professional musicians, and non-musicians in a visuospatial working memory task measured with the Spatial Span of the Wechsler Memory Scale, Third Edition.

In summary, while evidence exists regarding the association between musical practice and visuospatial abilities, the findings remain contradictory, and there is no clear consensus on the specific musical tasks that most significantly influence the development of these skills. Nonetheless, the literature suggests that spatial analysis is crucial for reading music due to the need to encode musical information and engage in cross-modal and intermodal integration processes [21,31,32,33,34]. Substantial evidence from eye-movement studies further supports the notion that printed music should be considered a visual stimulus despite being comparable from many perspectives to a verbal text-reading task [35,36,37]. Additionally, imaging studies demonstrate that spatial information is processed during music reading, activating brain regions specific to this task, such as the left inferior parietal lobe and the right posterior fusiform gyrus [38,39,40,41]. If music practice indeed enhances visuospatial cognition, further research is needed to identify which specific musical activities contribute most to the differences in spatial working memory between musicians and non-musicians.

### 1.2. The Corsi Block-Tapping Test

The Corsi block-tapping test (CBT) [42,43] was developed as an adjunct to the verbal memory task to measure hemispheric lateralization in epileptic patients. The CBT has been widely used to measure short-term memory in clinical cases with different progressive etiologies [44,45], as well as in those with traumatic brain injury, dementia, Parkinson’s disease, children with special learning needs, mental retardation, and other neurological dysfunctions. It has also been applied to a wide range of populations in both adults [46] and children [47] as part of the major neuropsychological battery [48]. The CBT relies on the sequential memory of locations engaging spatial attention [44]. Spatial working memory can be estimated by increasing the length of the sequences [49,50,51].

The findings obtained from the CBT, particularly as an indication of spatial memory, are being debated and challenged [52]. Some researchers have argued that the CBT involves executive processes [53,54,55,56]. Moreover, visuospatial and executive processes seem to share a similar cognitive architecture [54,55] or at least to be more closely related than verbal and visual short-term processes [55,57]. According to Berch et al. [58], the CBT task may measure more than just visuospatial processing due to its sequential organization. Moreover, the research addresses the question whether the underlying mental operation of the task could be interpreted as an amodal spatial attention mechanism or a type of visual imagery. More recent findings show that the CBT implies not only visual analysis but also planning processes, as observed with eye-tracking protocols [59].

Some previous studies have reported differences between musicians and non-musicians on the CBT task [32,60,61], while others found no differences [34,62]. Furthermore, it is not clear how to interpret these results because, as previously mentioned with reference to music reading, a standard criterion for assessing musical background is not usually defined. For example, Amer et al. [60] claimed that the more robust differences found in their study were related to the inclusion of professional musicians who had more training and expertise than amateur musicians. Additionally, Giovagnoli and Raglio [62] point out the importance of considering specific competences associated with a particular degree of musical education. Other works focus on other factors, such as the number of years (minimum 5) of musical training [34]. Roden et al. [63] found no differences in the CBT task between two groups of primary school children: those in music training and those in natural science training. The children in music training received 45 min of training per week for a period of 18 months, consisting of a variety of activities such as singing, clapping, and pitch identification exercises. In older populations, Grassi et al. [64] reported no differences between musicians and non-musicians in the forward and backward version of the CBT (declared by the authors as a short-term memory task). However, they found differences in other visuospatial tasks (the short Embedded Figures Test and the short Mental Rotations Test), where musicians presented higher scores. Something similar occurred with complex working memory tasks (i.e., Visual Pattern Test Active). The authors concluded that musicians’ cognitive reserve could mitigate the effects of age-related cognitive decline, as long-term musical training is thought to enhance neuroplasticity.

The neural correlates of visuospatial working memory assessed through the CBT have been explored in a limited number of neuroimaging studies utilizing both the original and computerized versions of the task. According to Lancia et al. [65], while there is growing evidence supporting the involvement of the prefrontal cortex, there is less consensus regarding the role of its subregions. Their findings, based on functional near-infrared spectroscopy (fNIRS), did not clearly demonstrate the roles of the dorsolateral prefrontal cortex (DLPFC) and the ventrolateral prefrontal cortex (VLPFC) in performing the standard CBT or in the block suppression test paradigm developed by Beblo et al. [66], which requires inhibiting responses to distractor cubes and involves a higher level of executive control. In contrast, Toepper et al. [67], employing functional magnetic resonance imaging (fMRI), observed the activation of specific areas such as the DLPFC and the VLPFC. They interpreted the activation of the VLPFC as crucial for updating working memory content and inhibiting visuospatial distractions. More recently, Panico et al. [68] utilized fNIRS with the classical wooden board version of the CBT and reported that the activation in the prefrontal cortex increased with cognitive load, suggesting that the demands of the task significantly influenced neural engagement.

### 1.3. Age and Sex Differences in Visuospatial Working Memory

Age has been described as a factor influencing performance on working memory tests. Considering Baddeley’s working memory system components, age differences could depend on the task content (i.e., verbal or visuospatial) [45,69,70]. Moreover, performance has been shown to be impaired in late adulthood [71]. Age has a detrimental effect on processing capacity but does not have the same effect on storage capacity [72,73,74].

Age differences in spatial working memory have also been found using the CBT by manipulating the presentation sequence. If the set size is shorter at the beginning and gradually increases in length, the format is ascending; otherwise, it is descending; the CBT has traditionally been used in ascending format. Rowe et al. [75] tested older and younger participants in two experiments by administering computerized versions of a 3 × 3 matrix and an electronic version of the CBT in ascending and descending formats. The authors showed that the effects of age (age–condition interaction) were determined by the disruptive effect that earlier information had on the retrieval of the most recent information. This effect is called proactive interference; it can negatively influence long-term memory retrieval and accrues over trials [76]. Kessels et al. [45] found significant correlations, albeit small, between the descending format and age. However, the authors found no significant differences between the forward and backward versions of the test, suggesting that both versions lie in the same cognitive processes. The backward version consists of the inversion of recall of the sequences by starting with the last block and finishing with the first. There is evidence that this procedure engages central executive functioning [45,77,78]. Moreover, Vandierendonck et al. [56] demonstrated, with a sample of twenty-five first-year university students, that the forward and backward recall of the CBT lie in central executive processing. Moreover, it was shown that a single training session of CBT protocols may influence mental rotation abilities in older adults [79].

Sex differences in working memory tests have also been well documented as a cognitive ability [80] and a domain-specific factor in working memory [81]. The significance of sex differences in visuospatial ability is constrained by task-specific factors [82,83,84]. For a detailed literature review considering task-specific factors (i.e., spatial memory, spatial rotation, navigation, object location), see Andreano and Cahill [85]. The authors confirmed the predominant opinion of a male advantage in spatial tasks and a female advantage in verbal tasks. They concluded that sex differences are noted in specific tasks engaging mental representation of space, especially when spatial information is the “primary available cue”. Kaufman [81] found sex differences in spatial working memory tests but not in verbal working memory tests, suggesting that working memory can be fractionated. This is supported by evidence of the specialized involvement of different brain structures in spatial memory [86,87,88]. As pointed out by Andreano and Cahill (2009) [85], those different components can be neuropsychologically dissociated [89], showing that spatial memory can be considered a multidimensional concept rather than a unitary function [50]. Indeed, spatial ability components involve different skills [84]. Kaufman [81] demonstrated that the nature of the processing component produces a small male advantage, which is consistent with the meta-analyses of Linn and Petersen [90] and Voyer et al. [84]. More recently, Tascon et al. [91] reported a male advantage in three different visuospatial tasks assessed under varying conditions, including different levels of difficulty and the active or passive involvement of participants. They suggested that the basis for these differences lies in the ability to use allocentric reference frames, accuracy in creating cognitive maps, mental rotation skills, and visuospatial span capacity.

Some authors have reported sex differences on the CBT [92,93,94,95], while others have not [45,50,96]. Sex differences are more easily detected in large population samples; in other words, from a cognitive point of view, those differences are not significant [51]. In samples of 495 subjects [97] and more than 1000 subjects, both adults and children [98,99], superior performances by men were reported (albeit with small effects).

It is important to note that some studies have challenged the idea about the cognitive mechanisms underlying the processing of spatial sequences in the computerized vs. physical versions of the CBT task. Computerized versions of the task can be compared (analogous) to physical versions in terms of average span and error rates [92]. However, there is evidence that the forward and backward versions of the task may differ between traditional and computerized versions. Claessen et al. [100] examined recall accuracy in both versions of the task in university students and reported that backward performance was similar; in the forward computerized condition, the responses were less accurate in comparison to the standard version. The authors suggested that the traditional version of the task led to motor priming effects linked to the movements of the experimenter. A more recent review of modern methodological practices regarding the CBT by Arce and McMullen [101] highlighted that since the introduction of the first digital formats of the CBT, and even earlier with traditional formats, the standard methodology was frequently either not adhered to or not documented. This included minor changes such as block color or positioning, which are often omitted from reports. The authors emphasized the importance of developing a standardized digital version with open-source code, as this could enhance the reporting and analysis of perceptual issues related to spatial memory.

### 1.4. Metacompetences, Metacognition, and Visuospatial Working Memory

The concept of metacompetence offers a valuable framework for understanding the awareness of personal abilities, with several theoretical implications. Leplat [102] argued that metacognition and metaknowledge are integral to the notion of metacompetence, as both contribute to task execution and share an operational nature. Metacognitive skills involve self-awareness and evaluation of cognitive processes [103], while metaknowledge pertains to knowledge about knowledge or cognition itself [104]. Wittorski [105] further expanded on this by positioning metacompetence within the realm of reflective practice, suggesting that skills generate metacompetence through the management of action capabilities. Additionally, the ability to self-monitor and regulate one’s own performance is encompassed within the construct of metacompetence [106]. The notion has been extended to research on scientific problem-solving in children, introducing the idea of meta-representational competence, which diSessa [107] defined as the ability to create, select, and understand the most appropriate representations for solving specific problems.

Metacognition in experimental tasks is frequently assessed by examining the statistical relationship between participants’ confidence judgments and their actual performance, a concept known as metacognitive sensitivity [108]. In the context of working memory, similar principles apply, with research suggesting that individuals tend to overestimate their performance through confidence judgments while simultaneously underestimating the magnitude of their errors [109]. This tendency is particularly pertinent to judgment of learning (JOL), a specific type of metacognitive judgment that involves forecasting future memory performance. JOL can be evaluated through two key dimensions: resolution, which measures the ability of JOLs to distinguish between items, and calibration, which assesses the accuracy of JOLs in predicting overall performance levels [110].

Research suggests that while there is some alignment between subjective judgments and objective measures of visual working memory, subjective judgments do not always accurately reflect visual working memory content [109]. For this, a general metacognitive architecture should be considered with a similar cognitive architecture supporting visual perception and visual short-term memory [111]. Adam and Vogel [112] demonstrated that while subjective judgments may predict certain variations in memory performance, participants consistently failed to recognize their own memory errors. Furthermore, Fleming [108] argued that metacognitive judgments are often inferential and can diverge from actual task performance.

Evidence from structural and functional brain imaging studies demonstrates an association between metacognitive abilities and task execution, supporting the existence of both domain-specific metacognitive processes and a domain-general metacognitive architecture when relevant stimulus features are shared across domains [111]. Indeed, despite the well-established influence of the prefrontal cortex [113], there is growing evidence that other brain regions may also contribute to metacognition in memory tasks, including functionally distinct metacognitive systems [114]. Furthermore, the connection between the prefrontal cortex and these other regions, such as the precuneus and the ventral striatum, is considered essential [108,115]. In the domain of listening behavior, Alavash and Obleser [116] highlighted the presence of two distinct cortical network systems that contribute to individual variability in metacognitive abilities. These networks, related to auditory processing (sensory network) and attentional control, appear to function independently yet interactively, shaping the substantial differences in the metacognitive performance observed across individuals.

Metacognition is a fundamental component of both music learning and performance, as it underpins the ability of professional musicians to engage in effective self-regulation and optimize their practice strategies [117,118,119]. Music reading engages visuospatial processing skills, as musicians must visually decode notation while simultaneously preparing for the physical execution of notes. This dual demand may be enhanced by the metacognitive skills that musicians develop over time, fostering effective anticipation and self-regulation during music reading. High levels of metacognitive skills among musicians have been shown to influence JOL and facilitate more efficient learning processes in performance contexts [120]. Notably, research has demonstrated that musicians differ from non-musicians in their metacognitive judgments concerning auditory scenes. Musicians tend to exhibit greater awareness of ambiguity, an effect strongly correlated with years of formal training, which suggests that these differences may stem more from perceptual mechanisms rather than decisional processes [121].

To summarize, the association between metacompetence and music training is inherently tied to the metacognitive abilities cultivated by musicians. Given that this study focused on a particular subset of operational competencies in musicians—namely, the visuospatial skills required for proficient music reading—we adopted the term “metacompetence” as a more appropriate framework. This concept more accurately reflects the theoretical considerations outlined in the previous discussion.

### 1.5. Rationale for This Study

As previously noted, music reading involves the activation of neural networks associated with spatial processing [122] and implies a reliance on visuospatial abilities assessed by the CBT in certain musical styles [123,124]. Considering these factors, along with the role of executive functions in both musical and visuospatial skills—particularly in relation to the CBT and executive functions [52,55,125]—the aims of this study were as follows:

The first goal of this study was to explore the association between spatial working memory and music practice, particularly in the context of music reading. The literature reviewed suggests that musicians demonstrate a visuospatial advantage. Given that music reading engages the cognitive processes associated with spatial memory, examining this relationship could clarify whether differences in spatial working memory between musicians and non-musicians emerge as a function of musical experience.

The second goal was to examine whether participants were aware of these abilities and if this awareness was fostered through musical practice. The literature reviewed indicates that musicians tend to exhibit not only enhanced visuospatial skills but also higher levels of metacognitive awareness. By examining CBT performance alongside metacognitive awareness, we aimed to explore the relationship between cognitive processes, self-awareness, and music reading skills, acknowledging the potential influence of various individual, contextual, and musical-practice-related factors.

We hypothesized that musicians develop disciplinary metacompetence in music reading, which may enable them to better estimate their own visuospatial capabilities compared to non-musicians. We expected to find enhanced spatial working memory in musicians, as compared to non-musicians, after accounting for potential influences of sex and age.

For the sake of clarity, in this paper, when we refer to music training, we specifically mean musicians who are proficient in reading sheet music or tablatures. However, we use the term ‘music training’ more generally when referencing the consulted literature. In this context, the term ‘musicians’ refers to individuals familiar with written music traditions.

## 2. Materials and Methods

### 2.1. Participants

Seventy musicians and non-musicians from the Pontifical Catholic University of Valparaíso, the University of Chile, and the University of Burgundy participated in this experiment. The musician group consisted of people who had regularly practiced reading music in either scores or tablatures for at least one year. The mean age of the musicians retained in the final sample (*n* = 31) was 23.6 years (*SD* = 3.6, range = 19–35). The mean age of the non-musicians retained in the final sample (*n* = 29) was 24.62 years (*SD* = 6.17, range = 18–40). For the musicians’ group, the average number of years of experience in reading music was 8.25 (*SD* = 5.25, range = 1–25). A total of 52.5% of musicians declared to be women and 47.5% men. In the non-musicians’ group 52% declared to be women and 48% men. Participants were matched by age (*t* (58) = 0.83, *p* = 0.41, *d* = 0.21, 95% CI [−1.52, 3.66]) and sex (Mann–Whitney *U* test, *p* = 0.51). Considering the various perspectives on learning music reading, the manner in which musicians typically approach a musical score or tablature was not included as a criterion for inclusion or exclusion.

### 2.2. Stimuli

**Figure A1:**
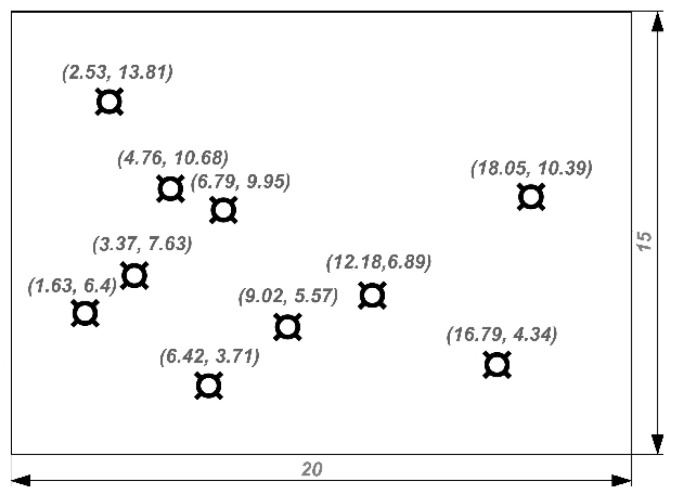
Corsi block-tapping test’s experimental display. The coordinates (in cm) are measured from the center of each figure.

The CBT procedure was applied on a computer. The presentation window measured 20 × 15 cm and included ten potential target locations presented as two-dimensional “currency sign blocks” of equal size (see Figure A1). Each block in turn was highlighted by changing its color to red for 1 s, with an inter-block time of 0.5 s. The CBT did not include an aural component.

### 2.3. Procedure

**Figure 1:**
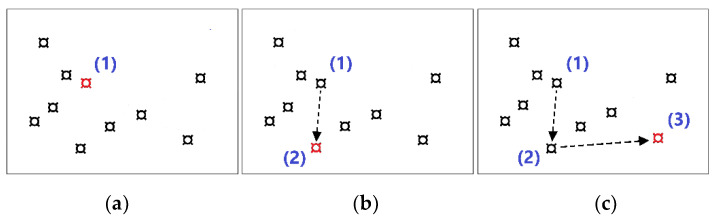
Example of a 3-block sequence in the CBT. The blocks light up in red in the order (1), (2), (3), as shown in panels (**a**–**c**). The task is considered correctly completed if the participant clicks on the blocks in the same order. However, if the participant does not recall the full sequence but clicks at least one block in the correct position within the sequence, such as (2), (1), (3) (e.g., panels **b**,**c**,**a**), the response would be considered partially correct, with the third block correctly identified. To advance to the next level, which involves a 4-block sequence, the participant must correctly complete the full sequence in at least one of three attempts.

At the beginning of the experiment, participants completed the CBT. Instructions and test materials were provided visually on a computer screen, with each trial initiating after a brief five-second preparation period. A sequence of highlighted blocks appeared in a randomized order, which participants were required to reproduce by selecting the blocks with a mouse. The initial sequences contained two blocks, gradually increasing by one block every three sequences until participants could no longer accurately recall the order. Progression to the next level was contingent on successfully completing at least one out of three attempts. The main variable measured was the total count of correctly recalled items in the exact sequence. Extra points were also given for individual items that were recalled accurately in terms of both order and location, regardless of whether the entire sequence was accurately reproduced. The final result was calculated based on the highest level attained by the participant using the formula: FinalResult = L − 1 + avLevelScore/L. In this context, L represents the highest level reached, and avLevelScore denotes the average score achieved at that level. The average score was calculated by dividing the total points awarded for correctly recalled items (including both order and location) by the total number of blocks presented. For example, if a participant at level 3 recalled the order and location of two blocks correctly in two attempts, and only one block correctly in the third attempt, the average score would be calculated as (2 + 2 + 1)/3 = 1.67. Consequently, the final result would be calculated as 3 − 1 + 1.67/3 = 2.56 (see Figure 1).

Following the CBT, participants attended an interview to discuss their musical background and assess their visuospatial memory abilities as ‘good’, ‘poor’, ‘normal’, or ‘unable to assess’. Additionally, they provided information about their age, the age they began reading music and practicing an instrument, their current field of study (if applicable), or their professional occupation.

It is important to note that for the purposes of this study, we opted not to include in the statistical analysis participants who were unable to assess their metacompetence (*n* = 2) or those who declared their abilities as ‘normal’ (*n* = 8). This decision was primarily made to maintain clearer distinctions in metacognitive self-assessment levels and to avoid imbalance in the statistical analysis, as the group with “normal” responses was relatively small compared to the other groups. However, certain qualitative aspects related to these participants were considered, particularly when discussing exceptional performance.

### 2.4. Data Analysis

Statistical analyses were conducted by treating music reading experience (minimum of one year) as a dichotomous variable to differentiate between musicians and non-musicians. The relationships between CBT performance and factors associated with musical training, such as years of music reading, were assessed using analysis of covariance (ANCOVA), controlling for the effects of sex and age. This control was implemented because prior studies have reported gender and age differences in CBT performance (see Section 1.3 “Age and Sex Differences in Visuospatial Working Memory”). A further one-way ANOVA was performed on the musicians’ group to determine whether enhanced spatial working memory capacities depended on years of music reading experience.

Prior to conducting the ANCOVA, homogeneity of variances was examined, and Levene’s test indicated no violation of equal variances (*F*(1,58) = 0.90, *p* = 0.77). Independence between the covariate age and the independent variable (groups) was confirmed (*F*(1,58) = 0.69, *p* = 0.41, η_p_^2^ = 0.001), as well as the assumption of homogeneity of regression slopes (*F*(1,56) = 2.35, *p* = 0.13, η_p_^2^ = 0.04).

## 3. Results

### 3.1. CBT Performance of Musicians and Non-Musicians

To determine the differences between the musicians and non-musicians in spatial working memory, a one-way analysis of covariance (ANCOVA) was performed, controlling for participant sex and age. A main effect of musical training was found (*F*(1,56) = 7.52, *p* = 0.008, η_p_^2^ = 0.118) on CBT performance and a reliable effect of the covariate sex (*F*(1,56) = 8.25, *p* = 0.006, η_p_^2^ = 0.128) but not age (*F*(1,56) = 2.17, *p* = 0.15, η_p_^2^ = 0.037). Musicians obtained significantly better CBT scores (*M* = 7.04, *SD* = 0.78, 95% CI [6.75, 7.32]) than non-musicians (*M* = 6.30, *SD* = 1.10, 95% CI [5.88, 6.72]), while the men scored higher (*M* = 7.04, *SD* = 0.92, CI 95% [6.69, 7.38]) than the women (*M* = 6.32, *SD* = 0.98, CI 95% [5.96, 6.69]). An additional ANCOVA, which treated age as a factor while retaining sex as a covariate, revealed that the effect of musical training remained significant (*F*(1, 56) = 8.67, *p* = 0.005, η_p_^2^ = 0.134). The reliable effect of the covariate sex was also maintained (*F*(1, 56) = 8.40, *p* = 0.005, η_p_^2^ = 0.131), and no main effect of age was observed *(F*(1, 56) = 3.12, *p* = 0.08, η_p_^2^ = 0.052).

### 3.2. Overall Correlations

The overall correlations between age and CBT performances were not significant (*r*(60) = −0.20, *p =* 0.14) nor were the correlations within groups (musicians and non-musicians) (*r*(31) = 0.14, *p =* 0.46, *r*(29) = −0.30, *p =* 0.11, respectively).

### 3.3. Metacompetences and Musical Training

**Figure 2:**
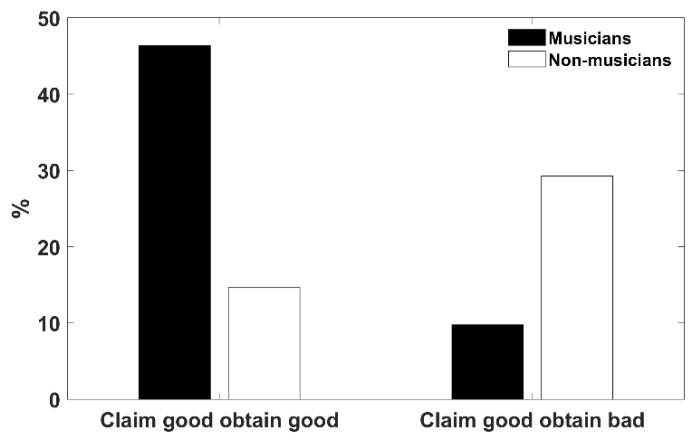
Self-evaluation of metacompetences in musicians and non-musicians. Bars represent the self-assessment of participants: left—claimed to have good visuospatial capabilities and obtained good performance on the CBT (musicians 46.34% and non-musicians 14.63%); right—claimed to have good visuospatial capabilities and obtained poor performances on the CBT (musicians 9.76% and non-musicians 29.27%).

We found that 59.2% of participants claimed to have good visuospatial capabilities, 26.3% stated that they did not have good visuospatial capabilities, 11.8% stated that they had normal capabilities, and 2.6% were not able to distinguish or evaluate their own capabilities. The accuracy in the self-evaluation of musicians and non-musicians was calculated from a multiple response table (see Figure 2). A chi-square analysis revealed that musicians demonstrated more highly developed metacognitive competencies compared to non-musicians (χ^2^ = 14.43, df = 4, *p* = 0.006).

After verifying the actual score obtained on the CBT according to the participant’s metacognitive accuracy, we observed that, considering the entire sample, there was no difference in the actual CBT scores between participants who claimed to have good visuospatial capabilities (*n* = 41) and those who declared they do not (*n* = 19) *(F*(1,58) = 0.342, *p* = 0.56, η_p_^2^ = 0.006). While the difference was minimal, it was not significant (CBT = 6.73 vs. 6.57, respectively). As previously stated, this difference became clearer when the groups were examined separately according to musical training (see Table 1).

### 3.4. Music Reading Experience and the Corsi Block-Tapping Test

The musician sample was divided into two groups according to the number of years of music reading experience: the first group was between 1 and 7 years (*n* = 16) (CBT scores, *M* = 7.24, *SD* = 0.73, 95% CI [6.85, 7.63]); the second group was between 8 and 25 years (*n* = 15) (CBT scores, *M* = 6.82, *SD* = 0.79, 95% CI [6.38, 7.25]). The one-way ANOVA yielded no significant differences between the groups in CBT performance (*F*(1,29) = 2.45, *p* = 0.128, η_p_^2^ = 0.078).

**Figure 3:**
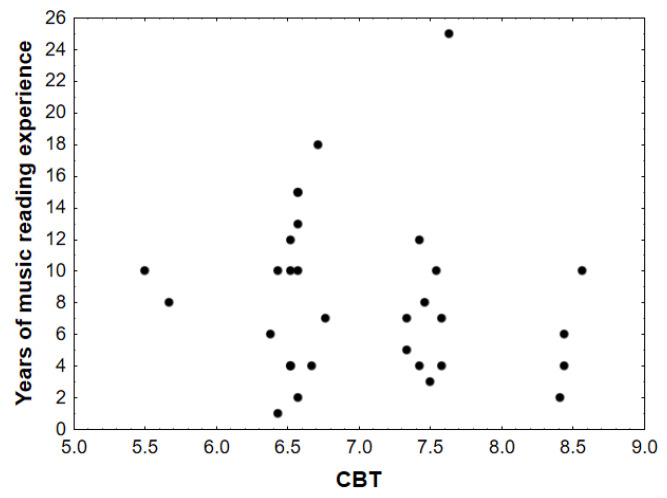
Results on the Corsi block-tapping test as a function of years of music reading practice: 54.84% of participants scored under 7 points (*M* = 6.44, *SD* = 0.34); 32.26% scored between 7 and 8 points (*M* = 7.48, *SD* = 0.10); and 12.9% scored over 8 points (*M* = 8.36, *SD* = 0.06). The black circles represent individual observations from different participants in the visuospatial task.

Moreover, no significant correlations were found between years of music reading and CBT score (*r* = −0.12, *p* = 0.51). These results suggest that enhanced spatial working memory in musicians may not be directly dependent on music reading experience (see Figure 3).

### 3.5. Metacomptences and Exceptional Performances

One participant scored exceptionally outside the norm. He was 20 years old, a non-musician, and played very few video games. He claimed to have normal capabilities of perception and visual memory and had an especially good memory for faces. This participant also developed spontaneous and quite complex strategies. In the first sequence (up to level 6), he remembered the blocks one by one. At level 7 he utilized a strategy of grouping in units of 3 + 3 +1. At level 8, he used a 2 + 2 + 2 + 2 grouping organization. At level 9, depending on shapes or paths of each sequence (i.e., curve or line), he followed the blocks. At level 10, he declared that the display became very complex, and he sometimes relied on quick short-term memory recall, which he found “more confusing”. He confirmed using another strategy, which consisted of isolating the more distant blocks and focusing on small, near trajectories.

It is important to note that the group reporting normal abilities (*n* = 8) obtained an average score of 7.38 on the CBT, while the two participants who reported uncertainty regarding their abilities, both of whom were musicians, had a higher average of 8.09. Among those declaring normal abilities, four were non-musicians, and two were musicians. This pattern suggests a potential trend that may warrant further investigation, particularly concerning the perception and assessment of metacognitive competencies in musicians.

## 4. Summary and Discussion

In the present study, we investigated whether music training is linked to enhanced spatial working memory, considering musicians with different levels of music reading experience. After covarying for age and sex, we demonstrated that musicians had better visuospatial capabilities and metacompetences than non-musicians, confirming our first hypothesis. While the development of this awareness occurs through specific experiences beyond the scope of this work, it is important to note that the process of making these competences functional often becomes fairly automatic [102]. The absence of an association between years of music reading and CBT scores refutes our second hypothesis and raises questions about the influence of music reading on the development of visuospatial capacities (transfer effects). In parallel, sex—but not age—influenced the observed differences between groups in the CBT. In focusing on music reading activities rather than broader measures of musical ability, such as musical sophistication [126,127], this study emphasizes a specific dimension of musical experience associated with visuospatial and cognitive skills, distinguishing it from the more general aspects of musical engagement.

### 4.1. Retrospective Self-Evaluation and Learning Strategies

Our results regarding participants’ self-evaluation of their visuospatial capacities suggest that metacognitive accuracy is primarily attributable to their specialized cognitive background. This finding contributes valuable insights to the current research, as the literature provides limited clarity on the influence of cognitive background on metacognition concerning spatial working memory, particularly in relation to participants’ professional specialization. Accordingly, our choice to consider these confidence predictions as professional metacompetences aligns with the theoretical framework presented in the Introduction. Moreover, as highlighted by Boldt [128], individuals tend to develop confidence predictions that are shaped by previous experiences of confidence, which can be linked to the heuristic approach in the formation of metacognitive judgments [129,130,131]. One possible interpretation of our results is that these aspects likely evolve dynamically alongside the visuospatial specialization that accompanies musical activities, particularly in the context of music reading.

Conversely, overconfidence was predominantly observed among non-musicians, consistent with previous findings in students aged 18–35 [109]. This raises the question of whether this lack of personal awareness may have influenced their performance on the CBT. Hoffman and Schraw [132] suggest that self-efficacy becomes particularly beneficial as working memory demands increase. It is likely that the non-musicians in this study lacked a clear understanding of the connection between the CBT and visuospatial abilities. In contrast, musicians appeared to possess a more developed awareness of this relationship.

This raises the question of whether the metacognitive knowledge musicians possess about their visuospatial advantages contributes to the development of more effective self-regulation strategies. Varela et al. [133] argued that musicians tend to display higher levels of metacognitive awareness, which is critical for shaping self-regulation strategies (see [117] for a review). In this context, it is plausible that musicians undergo a dynamic process that cultivates enhanced metacompetences, potentially leading to more sophisticated self-regulation mechanisms, as proposed by Boekaerts’ [134] Dual-Processing Self-Regulation Model. According to Samaha and Postle [111], adaptive behavior hinges on the capacity for accurate introspection regarding one’s own performance. However, as we explore in the next section, these cognitive profiles may depend on a range of factors that are not easily identifiable, and an exceptional case observed in the CBT results highlights how self-regulatory capacities can vary significantly depending on individual contexts.

In the case of the exceptional performance (CBT = 9.4) reported in this study and considering the findings of Vandierendonck et al. [56], the verbalized strategies declared seem to be related to the use of verbal support. We believe that given the exceptional nature of this performance, such an interpretation could be plausible, despite the fact that the authors cited observed verbal support in reversed-order CBT presentation. According to Baddeley [2], when high demand is placed on the working memory system, recall requires greater control. However, this assumption implies that both subsystems (verbal and visual) must be storing information independently [135]. Furthermore, the exceptional participant applied different strategies depending on the set size and the resulting block shapes. This is consistent with Brunetti et al. [92], who indicated that the subject’s responses can be planned during sequence presentation. Our participant also declared the use of grouping strategies, consistent with studies on sequential learning, reporting strategies of grouping three or four elements into one chunk [136,137]. With respect to his continuous adaptation of his strategies in response to increased set size, there is evidence suggesting that shifts in attention may interfere with the recall of a movement sequence [138,139]. Furthermore, the extent of disruption is notably less than that caused by comparable eye movements [82]. This can explain the need to use different strategies throughout the sequences.

### 4.2. Music Reading and Spatial Working Memory Capacities

The absence of a significant association between CBT performance and years of music reading experience can be interpreted from several perspectives. First, it invites consideration of previous research that suggests a relationship between musical practice and visuospatial working memory (see the Section 1). This highlights the possibility that multiple neuroplastic processes may be involved, which are not solely dependent on the systematic practice of a specific task, such as music reading. Music practice engages a complex cognitive system, and music reading itself is a multitasking activity, making it difficult to isolate potential transfer effects, particularly given the limitations of this study’s design. As Bigand and Tillmann [11] noted, controversial evidence surrounding the far transfer effect may stem from a methodological conflation of far and near transfer. In this context, it may be more critical to focus on how visuospatial skills are engaged during tasks like music reading rather than on the amount of working memory capacity. The effective mobilization of these skills could play a more significant role in performance than memory capacity alone, particularly in tasks that require complex cognitive integration [123]. The current findings align with this perspective, suggesting that the visuospatial demands of music reading can benefit from enhanced spatial memory. Musicians’ CBT performance, combined with their metacognitive abilities, may therefore reflect the cognitive integration required for accurate and efficient music reading.

It is also important to note the potential influence of context on the results, as the musicians in this study came from various countries with diverse music learning traditions. In Chile, many music students only begin reading music as adults during their university studies [140], whereas in France, there is a tendency to start music education earlier alongside formal training; this trend began to balance out in 2018. The age gap between amateur musicians significantly narrowed, with older participants increasing and younger ones declining in practice [141]. Consequently, many studies conducted in various countries assume vastly different age windows, with the onset of music practice often occurring before the age of 10 (e.g., [32]).

Furthermore, we observed that the less-experienced music readers showed variable profiles in common with those with more experience in their CBT outcomes (see Figure 3). Trainor et al. [142] analyzed EEG recordings and noticed a link between music practice and the development of executive functions. The authors showed that musicians have larger responses to gamma-band frequencies than non-musicians in groups of the same age range (three groups of adults and a group of 4.5-year-old children). Gamma-band activity within the visual domain is thought to play a role in integrating features like location, color, and shape into a unified conscious perception of an object [142,143,144]. From this point of view, and consistent with the findings reported here, the observed similarities in spatial working memory profiles among musicians with varying levels of experience further suggest that early exposure to music may positively influence the development of these capacities.

However, we cannot affirm that continuous practice of reading music entails a progressive increase in these capabilities. For example, Gagnon and Nicoladis [32] reported significant differences between musicians with at least six years of instrumental practice and non-musicians. Similarly, our study identified significant differences among musicians with a minimum of one year of music reading practice (mean = 8.25), reinforcing the argument raised in the Section 1 about the lack of consensus in defining critical details in the literature such as the number of years of music reading practice.

Another possibility for the mobilization of visuospatial capabilities, as mentioned in the previous section, relates to the development of competencies for reading certain styles of music, such as contemporary music, which may demand greater use of spatial working memory [123]. Additionally, specific music styles or repertoires may contribute to the development of visuospatial capabilities, though this requires further investigation. Notably, a recent study by Drai-Zerbib et al. [145], using a machine learning approach, showed that CBT outcomes can predict lower levels of expertise in music reading, particularly for classical music. See Perra et al. [146] for recent insights into this topic.

Moreover, it is important to acknowledge that the observed differences in spatial working memory or visuospatial self-assessment may be influenced by a range of factors, including personality traits, cognitive variables, or even emotional influence on spatial cognition [147,148]. Traits such as focus and perseverance may have impacted both CBT performance and participants’ decisions to engage in music learning. Thus, while musical practice may contribute to improvements in visuospatial capabilities, it is essential to recognize that these abilities are also shaped by a broader array of individual characteristics and experiences.

### 4.3. Influence of Age and Sex Covariates on Spatial Working Memory Differences

Our results suggest that experience with music practice may influence the visuospatial capabilities in musicians, regardless of their stage of formal training, as indicated by the absence of a main effect of age as a covariate. This finding aligns with research showing that sustained engagement in complex activities, like music, can support spatial working memory and executive functions in adults [9,64,149,150,151]. While age-related declines in visuospatial working memory typically begin around age 31 in women and age 40 in men, with effects influenced by task difficulty [152], these trends extend beyond the age range in our sample. As such, these observations require further investigation to determine whether music practice can help maintain visuospatial functions across a broader range of ages.

Considering the entire sample (both groups), our results are not consistent with previous research establishing age differences in spatial span [92,97,99,152]. However, the findings should be interpreted cautiously due to the differing age range of participants in the studies, and because age differences may also depend on the format in which the CBT is administered or on the influence of recent distractions, which may lead to proactive interference [75].

Concerning sex, our analysis revealed a significant main effect as a covariate, with men outperforming women in spatial working memory performance. Considering the points discussed earlier, these findings highlight the potential of musical training to influence spatial working memory, even when accounting for sex differences. While previous studies have reported a male advantage in visuospatial skills (see Introduction), evidence suggests that certain types of training, such as music or even video games, may help reduce sex disparities in spatial performance [153]. However, other factors, such as individual differences in experience and strategy use, may also contribute to this observed advantage.

The limitations of the present study are associated with the variability in the different music reading practices as well as the different musicians’ cognitive profiles. As in other previous studies, we should apply measures of general intelligence [61,154] or verbal ability [60] to control for these different cognitive profiles. Another limitation is that the whole sample consisted of participants from different countries as well as different cultural backgrounds. All of these factors can influence the development of visuospatial capabilities. Despite the potential influence of the CBT results on the metacompetence reports, it is important to note that participants were not fully aware of their performance on the test. The feedback provided is limited to a final score, allowing no possibility of direct comparison with other participants’ scores. This approach mitigates the impact of performance feedback on self-assessment, ensuring that participants’ reports of their visuospatial capabilities remain independent of their CBT results. Moreover, considering that the influence of metamemory could be viable, as suggested by Finn and Metcalfe [155], past test performance may lead to a match between memory and metamemory. This study did not employ strict inclusion and exclusion criteria defined by participants’ health conditions or medication use, as we sought to capture a broad representation of real-world cognitive performance among musicians and non-musicians. While severe psychiatric conditions were excluded, individuals who experienced mild anxiety or took common medications were included. Although this approach increased this study’s ecological validity, certain health factors may have subtly influenced cognitive performance. Future studies should control for these variables to better isolate the effects of musical experience on visuospatial working memory. Additionally, a more detailed assessment of different types of musical practices could provide insights into their nuanced effects on cognitive measures such as CBT performance.

## 5. Conclusions

We conclude that the CBT is effective for comparing visuospatial capabilities between musicians and non-musicians, revealing that visuospatial metacompetences are more highly developed in musicians. Our results suggest that the differences in spatial working memory between these groups cannot be attributed solely to time spent on music reading; rather, they may relate to enhanced executive functions in musicians, which supports the idea of a broadly applicable metacognitive framework. Drawing on insights from existing research [123,145], it is plausible that these visuospatial advantages vary according to the type of musical task or relate to familiarity with specific musical styles. While the potential for transfer to other cognitive domains remains an area for further exploration, our findings highlight the potential of musical practice in shaping both cognitive functions and metacognitive competencies, while acknowledging the possible contribution of factors beyond musical experience alone.

## Figures and Tables

**Table 1:** Descriptive statistics for different groups and metacomptences on the CBT.

Participants	Metacomp	Mean	SD	*LL*	*UL*	*n*
Non-musicians	Claim bad	6.35	0.95	5.71	6.99	11
	Claim good	6.27	1.20	5.67	6.87	18
	Total					*29*
Musicians	Claim bad	6.87	0.76	6.23	7.5	8
	Claim good	7.10	0.79	6.75	7.44	23
	Total					*31*

Note. *LL* = lower limit 95% confidence interval; *UL* = upper limit 95% confidence interval; *n* = sample number; Metacomp = metacompetences.

## Data Availability

The raw data supporting the conclusions of this article will be made available by the author upon request.
